# Targeted insertion of conditional expression cassettes into the mouse genome using the modified *i*-PITT

**DOI:** 10.1186/s12864-024-10250-0

**Published:** 2024-06-05

**Authors:** Hiromi Miura, Ayaka Nakamura, Aki Kurosaki, Ai Kotani, Masaru Motojima, Keiko Tanaka, Shigeru Kakuta, Sanae Ogiwara, Yuhsuke Ohmi, Hirotaka Komaba, Samantha L.P. Schilit, Cynthia C. Morton, Channabasavaiah B. Gurumurthy, Masato Ohtsuka

**Affiliations:** 1 Department of Molecular Life Science, Division of Basic Medical Science and Molecular Medicine, Tokai University School of Medicine, Kanagawa, Japan; 2 Life Science Support Center, Tokai University, Kanagawa, Japan; 3 The Institute of Medical Sciences, Tokai University, Kanagawa, Japan; 4 Department of Innovative Medical Science, Tokai University School of Medicine, Kanagawa, Japan; 5 Division of Hematological Malignancy, Institute of Medical Sciences, Tokai University, Kanagawa, Japan; 6 Department of Clinical Pharmacology, Tokai University School of Medicine, Kanagawa, Japan; 7 Departments of Basic Medicine, Tokai University School of Medicine, Kanagawa, Japan; 8 Division of Kidney, Diabetes and Endocrine Diseases, Okayama University Hospital, Okayama, Japan; 9 Laboratory of Biomedical Science, Graduate School of Agricultural and Life Sciences, The University of Tokyo, Tokyo, Japan; 10 Collaborative Research Institute for Innovative Microbiology (CRIIM), The University of Tokyo, Tokyo, Japan; 11 Research Center for Food Safety, Graduate School of Agricultural and Life Sciences, The University of Tokyo, Tokyo, Japan; 12 Department of Clinical Engineering, Chubu University College of Life and Health Sciences, Kasugai, Aichi Japan; 13 Division of Nephrology, Endocrinology and Metabolism, Tokai University School of Medicine, Kanagawa, Japan; 14 Program in Genetics and Genomics and Certificate Program in Leder Human Biology and Translational Medicine, Biological and Biomedical Sciences Program, Graduate School of Arts and Sciences, Harvard University, Cambridge, MA USA; 15 Harvard Medical School, Boston, MA USA; 16 Departments of Obstetrics and Gynecology and of Pathology, Brigham and Women’s Hospital, Boston, MA USA; 17 Institute Member, Broad Institute of Massachusetts Institute of Technology and Harvard University, Kendall Square, Cambridge, MA USA; 18 Manchester Center for Hearing and Deafness, University of Manchester, Manchester, UK; 19 Mouse Genome Engineering Core Facility, University of Nebraska Medical Center, Omaha, NE USA; 20 Department of Genetics, Cell Biology and Anatomy, College of Medicine, University of Nebraska Medical Center, Omaha, NE USA

**Keywords:** Targeted transgenesis, Conditional expression, PhiC31 integrase, FLP-*FRT*, Pronuclear injection-based targeted transgenesis, Mouse

## Abstract

**Background:**

Transgenic (Tg) mice are widely used in biomedical research, and they are typically generated by injecting transgenic DNA cassettes into pronuclei of one-cell stage zygotes. Such animals often show unreliable expression of the transgenic DNA, one of the major reasons for which is random insertion of the transgenes. We previously developed a method called “pronuclear injection-based targeted transgenesis” (PITT), in which DNA constructs are directed to insert at pre-designated genomic loci. PITT was achieved by pre-installing so called landing pad sequences (such as heterotypic *LoxP* sites or *attP* sites) to create seed mice and then injecting *Cre* recombinase or *PhiC31* integrase mRNAs along with a compatible donor plasmid into zygotes derived from the seed mice. PITT and its subsequent version, improved PITT (*i*-PITT), overcome disadvantages of conventional Tg mice such as lack of consistent and reliable expression of the cassettes among different Tg mouse lines, and the PITT approach is superior in terms of cost and labor. One of the limitations of PITT, particularly using *Cre*-mRNA, is that the approach cannot be used for insertion of conditional expression cassettes using Cre-*LoxP* site-specific recombination. This is because the *LoxP* sites in the donor plasmids intended for achieving conditional expression of the transgene will interfere with the PITT recombination reaction with *LoxP* sites in the landing pad.

**Results:**

To enable the *i*-PITT method to insert a conditional expression cassette, we modified the approach by simultaneously using *PhiC31o* and *FLPo* mRNAs. We demonstrate the strategy by creating a model containing a conditional expression cassette at the *Rosa26* locus with an efficiency of 13.7%. We also demonstrate that inclusion of *FLPo* mRNA excludes the insertion of vector backbones in the founder mice.

**Conclusions:**

Simultaneous use of *PhiC31* and *FLP* in *i*-PITT approach allows insertion of donor plasmids containing Cre-*loxP*-based conditional expression cassettes.

**Supplementary Information:**

The online version contains supplementary material available at 10.1186/s12864-024-10250-0.

## Introduction

Mice in which foreign DNA is inserted into the genome are called “transgenic (Tg) mice.” Since the development by Gordon *et al*. in 1980, many Tg mice have been produced by microinjection of DNA into fertilized eggs, and used for functional analysis of various genes and creation of disease mouse models [1]. Over the years, other methods have been developed to create Tg mice, including infection of early embryos with retroviral vectors and creation of chimeric mice from implanted modified embryonic stem (ES) cells. Although the microinjection method is quite simple, the genomic loci where the transgenes are inserted and their copy numbers are unpredictable [2]. Gene expression can be greatly affected by a position effect, by the state of chromatin at the insertion site, and by the regulatory sequences present in the flanking genomic sequences [3]. In addition, repeat-induced gene silencing may occur when genes with multiple copies are inserted in tandem, and thus there may not be a positive correlation between copy number and gene expression level [4]. Furthermore, some Tg DNA sequences may be subject to epigenetic effects such as DNA methylation [5]. Because reproducibility and stability of gene expression often cannot be obtained even within the same strain, it becomes necessary to analyze multiple founder lines of Tg mice to confirm that the phenotype is consistent.

This problem can be avoided by using ES cell-mediated gene targeting to insert a single copy transgene at a defined genomic locus (targeted transgenesis). However, the ES cell-mediated method is labor intensive, time-consuming, and expensive [2]. Since about a decade, CRISPR genome editing technology has been used to perform targeted transgenesis via microinjection technique. Even though CRISPR-based approaches are routinely used for generating conditional knockout- and short knock-in- models that require insertion of cassettes of about 1 to 2kb [6–9], these approaches are still inefficient for inserting cassettes of several kilobases long [6, 7, 10]. The DNA repair process often results in additional lesions such as short insertions or deletion (indel) mutations in addition to the target Tg allele [11–13], and the necessity of longer homology arms for plasmid-based inserts requires additional cloning steps. Other than these methods, targeted transgenesis methods using site-specific recombination and integrase systems derived from bacteriophages and microinjection have been developed by our group and several others [14–19].

We previously developed a modified transgenesis method called pronuclear injection-based targeted transgenesis (PITT). The first version of PITT relied on recombinase-mediated cassette exchange (RMCE) using the Cre-*LoxP* site-specific recombination system [14]. To generate targeted Tg mice using the PITT method, it is necessary first to generate a mouse strain with recombinase recognition sequences such as *LoxP* at a defined region of the genome. Although this prerequisite step is time-consuming and expensive, once a seed mouse line is established, there is no need to handle ES cells; many different types of targeted Tg mice can be generated using only direct microinjection of zygotes from the seed mice [2]. Because the seed mice will need to contain short landing pad sequences of only a couple hundred bases, such models can also be easily generated via a CRISPR approach that will obviate the need for any ES cell-based approaches [12, 20].

By using the PITT approach, we have generated a variety of Tg mice, including fluorescent gene-expressing mice [14, 21], tissue-specific gene-expressing mice [22, 23], and knockdown mice [14, 24], and have shown that transgene expression in these mice is highly reproducible and stable. Therefore, unlike Tg mice with randomly inserted transgenes, the mice generated by the PITT method have the advantage that multiple founder lines do not need to be generated, maintained, and analyzed.

Furthermore, we developed a seed mouse that allows use of multiple recombination systems, such as FLP-*FRT* and PhiC31 integrase, as well as Cre-*LoxP*. This modified PITT method was named improved PITT (*i*-PITT) and we demonstrated that simultaneous use of Cre-*loxP*-mediated recombination and PhiC31 integration significantly enhances the targeted insertion efficiency [15]. Although *i*-PITT system has a potential to insert a *LoxP*-flanked DNA cassette (commonly known as the ‘floxed cassette’) for the purpose of conditional expression, theoretically, this approach has yet to be demonstrated to generate conditional expression Tg mice. Specifically, transgene insertion by PITT using the Cre-*LoxP* system is not feasible for inserting a floxed cassette because the *LoxP* sites within the donor cassettes will be used up for Cre-mediated integration of the donor plasmid and therefore the recombined *LoxP* sites are unavailable for the conditional functionality of the transgene. To overcome this challenge, we devised an alternative strategy of PITT by using the PhiC31 and FLP-*FRT* systems.

## Materials and methods

### Mice

**Fig. 1:**
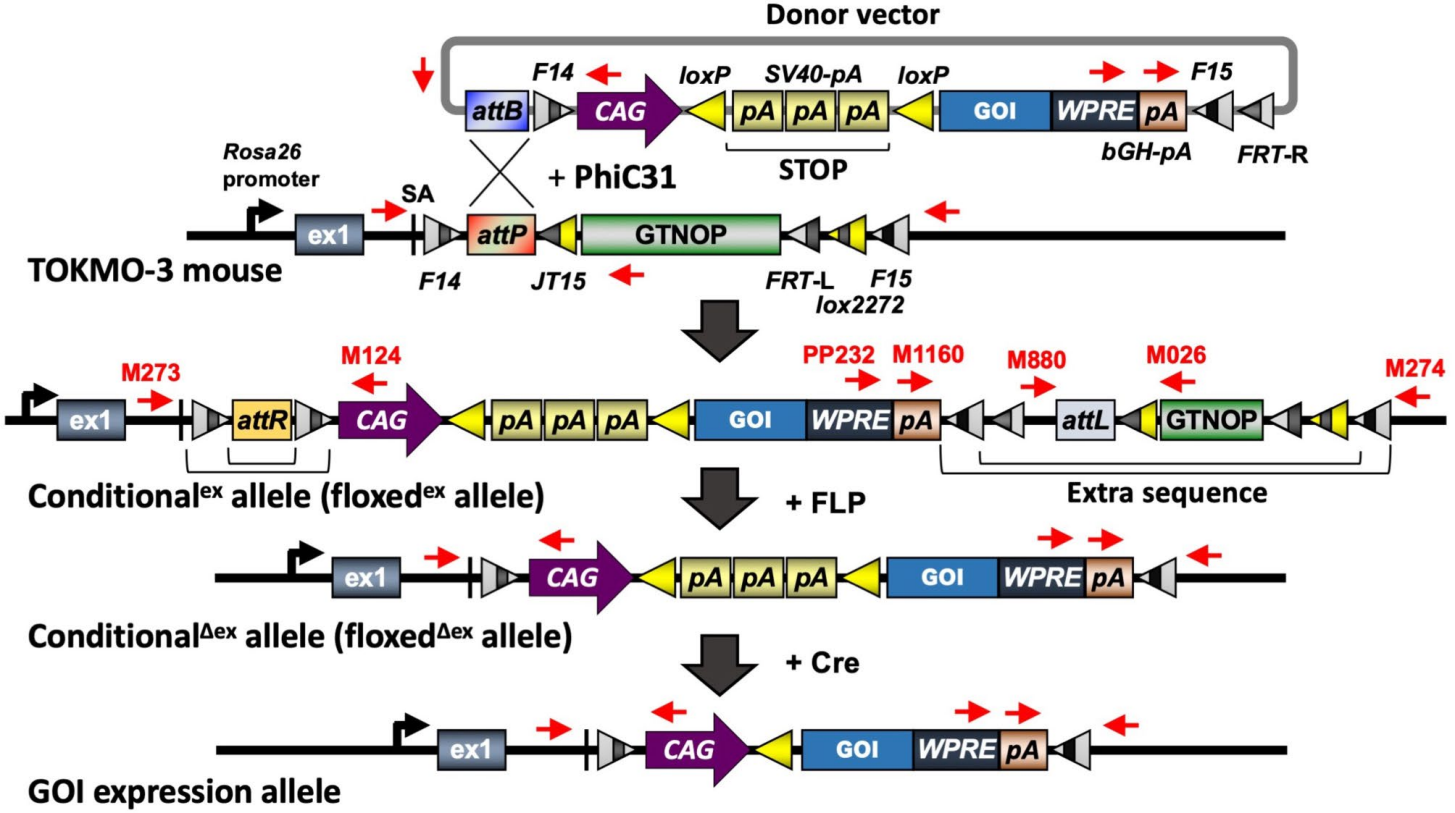
Schematic diagram of the insertion of a conditional expression cassette using the modified *i*-PITT method. Primers are shown in red (see Supplemental Table 1). The region shown as “STOP” consists of three *SV40-pA* sequences. *F14*, *F15*, *FRT*-L, *FRT*-R: mutant *FRT* sites. SA: splice acceptor. *WPRE*: woodchuck hepatitis virus posttranscriptional regulatory element. *Lox2272*: mutant *LoxP*. *bGH-pA*: bovine growth hormone polyadenylationsignal sequence. *SV40-pA*: Simian virus 40 polyadenylation signal sequence. GOI: gene of interest.*CAG*: hybrid construct consisting of the cytomegalovirus enhancer fused to the chicken beta-actin promoter. *attB*, *attP*, *attR*, *attL*: attachment sites for PhiC31 integrase. *GTNOP*: a cassette containing “*eGFP-T2A-Neomycin resistant gene-hOCT4-PolyA*” [15]

Inbred C57BL/6N and outbred MCH(ICR) mice were purchased from CLEA Japan Inc. (Tokyo, Japan). The seed mice (TOKMO-3) containing landing pads for targeted insertion of donor vectors were maintained as homozygotes with the inbred genetic background of C57BL/6N (Fig. 1A) [15]. NPHS2-CreERT2 mice (Tg(*NPHS2-cre/ERT2*)^Mkas^) [25] were mated with the Tg mouse conditionally expressing *Maff* (*Condi-Maff*; RBRC11275, generated in this study), to obtain *Condi-Maff/NPHS2-CreER*^*T2*^ mouse. The littermates containing only the *Condi-Maff* cassette were used as controls for assessing the conditional gene expression.

Mice were fed *ad libitum* under a 12:12 light and dark cycle, under the condition in specific pathogen-free (SPF). The animals were monitored daily, supplied with food. Non-transgenic mice were euthanized by cervical dislocation under anesthesia according to the Guidelines for the Care and Use of Animals for Scientific Purposes at Tokai University.

### Plasmid construction

Using commercial gene synthesis services (GENEWIZ) and conventional restriction enzyme-based cloning steps, pBIE, pBIK, and pBIM, plasmids containing a conditional expression cassette with *attB*, mutant *FRT*, *CAG* promoter, STOP cassette (three polyA addition sites flanked by *LoxP* sequences), *WPRE*, and polyA sequences were constructed (Supplementary Fig. 1). The three polyA sequences in the STOP cassette region are derived from the sequence of Ai65 plasmid (Addgene #61,577) [26]. Genes of interest (GOI) were inserted into restriction enzyme sites of these vectors to generate donor vectors 1 to 11 (DV1 to 11) listed in Table 1. For example, *Maff* cDNA was inserted into the pBIE vector to make plasmid DV4, which was used to generate a Tg mouse with conditional expression of the *Maff*. We used a donor vector without *WPRE* sequences in Project 11 (Table 1). The pBER donor vector containing a promoter-less tdTomato-polyA cassette was used to determine optimal concentration of *FLPo* mRNA [15]. With this system, tdTomato transgene is expressed from endogenous *Rosa26* promoter only when the pBER is inserted into the genome via PhiC31 integrase and/or FLP-*FRT* system.

**Table 1:** *i*-PITT experiments for integration of conditional expression cassettes using *PhiC31o* and *FLPo* mRNA

Project ID	Donor Vector (insert size) (vector size)	Zygotes injected	Zygotes transferred	Live born offspring obtained	Targeted integration (%)	Deletion of vector backbone*	Founder mouse ID
1	DV1 (4.6 kb) (6.9 kb) DV2 (4.8 kb) (7.1 kb)	280	216	27	4 (14.8)	2 (1)	#691,#696,#704,#707
2	DV3 (6.8 kb) (9.1 kb)	284	216	23	2 (8.6)	0	#547,#568
3	DV4 (4.3 kb) (6.6 kb) DV5 (5.5 kb) (7.8 kb)	241	219	11	1 (9.0)	0	#741
4	DV6 (8.7 kb) (11.1 kb)	150	141	22	2 (9.0)	0	#717,#720
5	DV5 (5.5 kb) (7.8 kb)	207	180	58	4 (6.8)	4	#883,#892,#15,#18
6	DV7 (5.0 kb) (7.3 kb)	173	160	20	2 (10.0)	2 (1)	#54,#56
7	DV8 (4.9 kb) (7.3 kb)	193	167	28	5 (17.8)	1	#62,#65,#68,#70,#82
8	DV1 (4.6 kb) (6.9 kb)	201	171	35	5 (14.2)	2 (1)	#107,#120,#121,#124,#130
9	DV9 (9.7 kb) (12.0 kb)	296	267	68	11 (16.1)	3	#2,#3,#11,#18,#31,#35,#40,#43,#44,#47,#52
10	DV10 (6.2 kb) (8.5 kb)	201	176	46	8 (17.3)	4	#180,#181,#186,#191,#212,#213,#219,#224
11	DV11 (3.3 kb) (5.6 kb)	214	195	50	9 (18.0)	6 (1)	#227,#233,#234,#257,#260,#265,#268,#270,#274
	Total	2440	2108	388	53 (13.6)	24 (4)	

* number of mosaic vector backbone-deleted mice shown in parentheses

### Preparation of mRNA

*PhiC31o* mRNA was used as previously reported [15]. The pBBJ plasmid (Addgene #62,672) used for generating *FLPo* mRNA was linearized using *Xba*I digestion, and *FLPo* mRNA was transcribed in vitro using mMESSAGE mMACHINE T7 Ultra Kit (Ambion) followed by purification of the mRNA using MEGAclear Kit (Ambion). mRNA was filtered by passing through an Ultrafree-MC filter (HV; 0.45 μm pore size; #UFC30HV00; Millipore) before mixing it with the donor plasmids [15].

### Microinjection

**Fig. 2:**
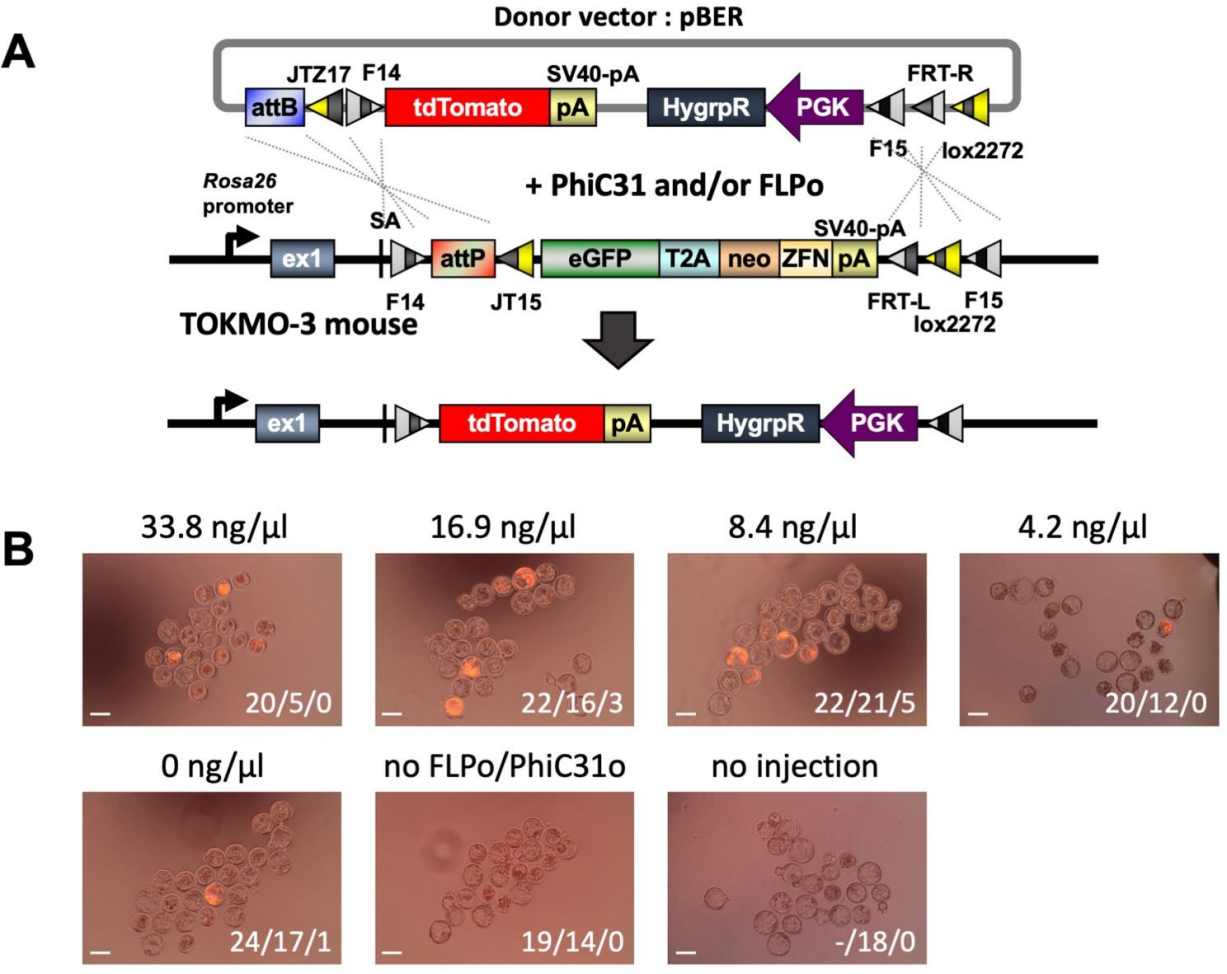
Fluorescence-based evaluation of targeted integration of donor vector into the *Rosa26* locus to evaluate optimal concentrations of *FLPo* mRNA. (**A**) Schematic diagram of the insertion of donor vector using the modified *i*-PITT method. The pBER donor vector DNA (containing a tdTomato-polyA cassette) and *PhiC31o* mRNA were used at 10 ng/ul and 7.5 ng/ul concentrations, respectively, along with different concentrations of *FLPo* mRNA (indicated above each image). Injections were performed at the zygote stage and red fluorescence was recorded at the blastocyst stage. (**B**) Fluorescence at the blastocyst stage. The numbers shown in the lower right corner of each photo are “number of zygotes that survived just after injection / number of embryos that developed to the blastocyst stage / number of normally developed eggs showing red fluorescence.” Scale bar: 100µm

In the experiments to generate Tg mouse strains, donor vector DNA(s) (5–10 ng/µl in total), *PhiC31o* mRNA (7.5 ng/µl) and *FLPo* mRNA (11.3 ng/µl) were mixed together in EmbryoMax Injection Buffer (#MR-095–10 F; Millipore). The DNA/mRNA mixtures were stored at − 80 °C until use. For removal of the donor vector backbone in founder mice, *FLPo* mRNA solution was prepared at a concentration of 15 ng/µl in EmbryoMax Injection Buffer. To determine optimal concentration of *FLPo* mRNA, the concentrations of the donor vector pBER (10 ng/µl) and *PhiC31o* mRNA (7.5 ng/µl) were kept constant, while the *FLPo* mRNA concentration was tested from 0 to 33.8 ng/µl (Fig. 2).

Unfertilized oocytes isolated from superovulated female mice (C57BL/6N) were subjected to in vitro fertilization (IVF) with spermatozoa obtained from a homozygous TOKMO-3 male mouse. Microinjection of the DNA/mRNA mixture was performed into both the pronuclei and cytoplasm of in vitro fertilized eggs. The injected embryos were cultured until the blastocyst stage to assess insertion efficiencies (by observing red fluorescence that originates from the inserted tdTomato) or transferred into the uteri of pseudopregnant MCH(ICR) females to allow for their development. Offspring were genotyped to assess successful targeted transgenesis. Injection of *FLPo* mRNA (15 ng/µl) into the cytoplasm of in vitro fertilized eggs derived from founder mice or the offspring was performed to eliminate the vector portion from *i*-PITT mice to obtain the conditional^Δex^ (floxed^Δex^) allele (without extra sequence) (Fig. 1).

### Detection of transgenes

**Fig. 3:**
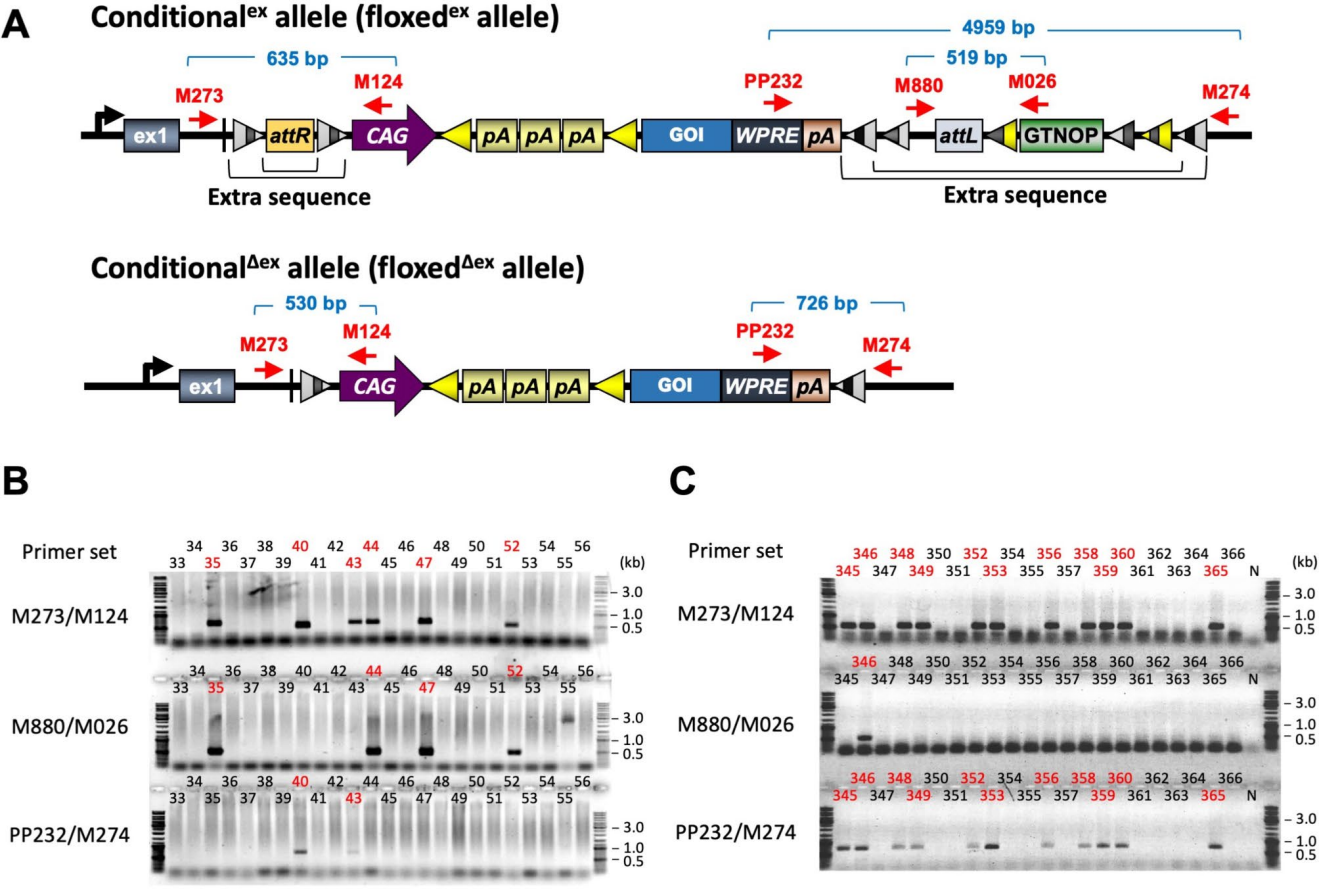
Examples of PCR genotyping analyses. Genotypes of each offspring (IDs indicated by numbers) were verified using three different primer sets (**A**). (**B**) A representative example of genotyping of offspring from Project 9 (Table 1). The PCR-positive offspring are indicated in red. (**C**) Example genotyping of pups obtained by injecting *FLPo* mRNA into fertilized eggs derived from offspring #56 with extra vector sequence obtained in Project 6 (Table 2). The PCR-positive offspring are indicated in red. N: Negative control. Full sized images and other raw data files relevant to this figure are included in Supplementary Information Figs. 2A-C and 3, and supplementary information files AT1054.TIF, AT1058.TIF and AT1061.TIF and AT0446.TIF

Correct insertion of donor vectors into the *Rosa26* locus, the site of pre-installed landing pad, was assessed by observing tissue samples under a fluorescence microscope and/or PCR-based genotyping of genomic DNA samples as described in Ohtsuka et al. 2015 [15]. For PCR detection of the transgene insertion in newborns, genomic DNA was isolated from the tail or ear using 40 to 50 µl of Allele-In-One Mouse Tail Direct Lysis Buffer (#ABP-PP-MT01500; Allele Biotechnology). PCR was performed in a total of 10 µl solution containing 2 x GC buffer I, 1 µl of the crude lysate, and the primer pair using TaKaRa *Taq*. For all experiments except 2 and 11, three primer sets viz. M273/M124 or M880/M026 or PP232/M274 were used for detection of targeted insertion allele, or targeted insertion allele with vector backbone, or detection of targeted insertion allele without vector backbone, respectively (Figs. 1 and 3A). For Projects 2 and 11, the GOI-specific and M1160 primers were used instead of PP232 primer (Supplementary Table 1). Nucleotide sequences of the junction were confirmed by sequencing.

### Conditional expression of *maff* transgene

To achieve podocyte-specific transgene expression of *Maff*, Tg mice with a conditional expression cassette for *Maff* (*Condi-Maff*; RBRC11275) were mated with NPHS2-CreERT2 mice (Tg(*NPHS2-cre/ERT2*)^Mkas^) [25]. Intraperitoneal injections of 75 mg/kg tamoxifen for five consecutive days were performed into the resultant *Condi-Maff/NPHS2-CreER*^*T2*^ mouse and littermate controls (*Condi-Maff* cassette alone) at 33-weeks of age. Kidney specimens were prepared nine weeks after the injections.

Conditional *Maff* transgene expression was detected by immunohistochemistry. Kidney tissues obtained from *Condi-Maff* Tg mice were embedded in OCT compounds, and 6 μm frozen sections were prepared. Sections were fixed in 4% paraformaldehyde for 10 min and incubated in PBS containing 0.1% Triton X-100. The following antibodies were used: rabbit anti-Maff (1;100, Protein-tech, 12771-1-AP) and goat anti-Nephrin (1:100, R&D, AF3159).

## Results

### Development of a system to generate tg mice for conditional gene expression

Transgene insertion using PhiC31-mediated integration alone does not allow subsequent removal of the extra vector backbone, and the backbone has prokaryote-derived sequences that can hinder reliable gene expression [14]. To solve these issues, we used a combination of the PhiC31 and FLP-*FRT* system, which could increase the insertion efficiency, and at the same time it allowed vector backbone deletion (Fig. 2A) [15]. The optimal concentration of *PhiC31o* integrase mRNA was previously standardized [15], and thus we examined the optimal concentration of only *FLPo* mRNA. The *FLPo* mRNA was set to final concentrations ranging from 0 to 34 ng/µl and mixed with the pBER donor vector carrying the red fluorescent gene tdTomato and 7.5ng/ul of *PhiC31o* integrase mRNA. The mixture was microinjected into the pronucleus and cytoplasm of fertilized eggs derived from TOKMO-3 mice [15]. After culturing embryos to the blastocyst stage, the success of donor insertion was examined by observing red fluorescence. In the first set of experiments, the insertion efficiency using PhiC31-mediated integration alone was low. For instance, of the 24 zygotes that survived just after injection 17 developed to blastocyst stage and only one of these showed red fluorescence, indicative of correct insertion) (Fig. 2B, Supplementary Table 2). In contrast, *FLPo* mRNA injection together with *PhiC31o* mRNA generated embryos showing red fluorescence when 8.4 ng/µl and 16.9 ng/µl *FLPo* mRNA were used (generated 5 and 3 red fluorescent blastocysts, respectively) (Fig. 2B, Supplementary Table 2). This suggest that the use of *FLPo* mRNA in combination with the PhiC31 system improves insertion efficiency compared to the experiments that used only PhiC31 integrase (1/24). Based on this result, we decided to use 11.3 ng/µl of *FLPo* mRNA, which is approximately in the midpoint of the two concentrations (8.4 ng/µl and 16.9 ng/µl), for all subsequent experiments, to generate live offspring.

The above experiment was repeated four more times to test if co-injection of *PhiC31o* and *FLPo* mRNAs produce consistent results. Even though we did not see statistically significant differences between *PhiC31o* alone or combination of *PhiC31o* and *FLPo* the insertion efficiency seems to be slightly higher when both mRNAs were injected (8/73 [11.0%, 0.0-23.5% in each experiment] in 33.8 ng/µl, 6/82 [7.3%, 0.0-18.8% in each experiment] in 16.9 ng/µl, 9/85 [10.6%, 0.0-23.8% in each experiment] in 8.4 ng/µl, 14/95 [14.7%, 0.0-23.8% in each experiment] in 4.2 ng/µl) than when only PhiC31 integrase was used (7/87 [8.0%, 4.5–12.5% in each experiment]) (Supplementary Table 2).

**Table 2:** FLP-mediated deletion of extra sequence containing vector backbone

Tg mouse lines used	Zygotes injected	Zygotes transferred	Live born offspring obtained	Offspring containing the transgene	Deletion of vector seq*
#704 from Project 1	131	120	20	6	4 (2)
#717 from Project 4	209	183	61	29	26 (8)
#56 from Project 6	155	140	22	11	11 (1)
#68 from Project 7	167	137	52	16	15 (2)
#130 from Project 8	156	150	34	15	9 (2)
Total	818	730	189	77	65 (15)

* number of mice with mosaicism shown in parentheses

Next, we designed and constructed several donor vectors for conditional gene expression (Supplementary Fig. 1). These cassettes enable conditional gene expression via the Cre-*LoxP* system. A target gene, downstream of a stop sequence (3 x *polyA*) placed between two *LoxP* sites, would be expressed after deletion of the stop sequence by the Cre-*LoxP* site-specific recombination. We generated a plasmid vector named pBIE (with *attB* and a pair of *FRT* sequences) that can insert the target DNA cassette (*CAG* promoter– *LoxP*– 3 x polyA– *LoxP*– *WPRE*– polyA) using the PhiC31 and FLP-*FRT* systems described above. Insertion cassettes included reporter genes such as *eGFP* and *mCherry* (pBIK and pBIM, respectively) as well as various GOIs.

### Insertion of conditional expression cassettes by the modified *i*-PITT method

Various vectors containing expression cassettes ranging from 3.3 to 9.7 kb (overall vector size from 5.6 to 12.0 kb) were mixed to a final concentration of 5–10 ng/µl, along with 7.5 ng/µl of *PhiC31o* integrase mRNA and 11.3 ng/µl of *FLPo* mRNA, and microinjected the solution into pronucleus and cytoplasm of fertilized eggs obtained from TOKMO-3 mice. Genotyping of pups obtained in a total of 11 projects revealed that founder mice with expression cassettes inserted at the *Rosa26* locus were obtained for all projects (Fig. 3A and B, Supplementary Fig. 2). The overall insertion efficiency was 13.7% (53/388) per live-born pups, and 2.2% (53/2440) per injected eggs (Table 1). Among the 53 pups that contained the targeted transgene, 24 (45.3%) had an insertion allele without vector backbone (termed as conditional^Δex^ allele) by FLP-*FRT* recombination. Four of the 24 pups (with conditional^Δex^ allele) also had conditional^ex^ allele containing vector backbone sequence, indicating that these mice were mosaic for both alleles.

We previously demonstrated that it is possible to obtain up to three different Tg mouse lines in one injection session by injecting several donor vectors simultaneously in the *i*-PITT method [15]. Two of our 11 different *i*-PITT experiments contained more than one donor vector (Table 1). Two out of the four pups obtained in project 1 (injected with DV1 and DV2 plasmids) had a cassette insertion of DV1, of which one of the mice also had a DV2 cassette at the same time. This indicates that one of the cassettes may have been inserted at a random genomic location or that the two cassettes were combined by intermolecular recombination via mutant *FRT* sequences and then inserted into the *Rosa26* locus by the PITT method. The other two pups had only the DV2 cassette inserted. Because the offspring with the DV1 cassette did not reproduce, another injection experiment with only DV1 was performed to obtain the desired Tg mouse line (Project 8). For the Project 3, only one offspring with a cassette of DV4 was obtained, and there were no offspring with DV5. Therefore, another injection experiment with only DV5 was performed to obtain the target Tg (Project 5). From these results, we conclude that although it may be possible to obtain multiple types of Tg founders in one injection session by mixing multiple vectors, performing individual injections to obtain multiple Tg lines may be more practical for *PhiC31o* and *FLPo* mRNA recombination.

### Example of incorrect junctional sequence of the donor constructs

**Fig. 4:**
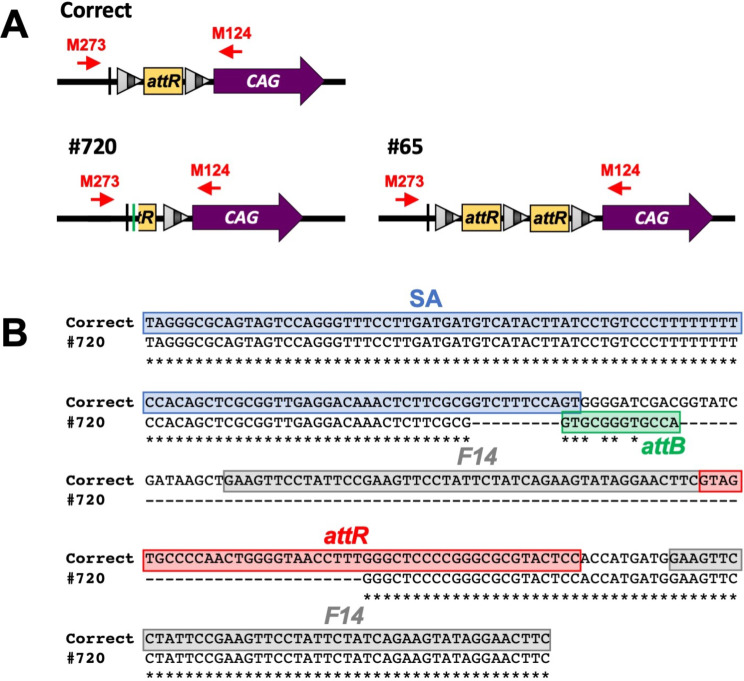
Example of incorrect junction sequences obtained after targeted integration. (**A**) The expected 5’ junctional architecture in the conditional^ex^ allele (floxed^ex^ allele) is shown as “correct”. Diagramed below are the incorrect 5’ junctional sequences identified in #720 (from Project 4) and #65 (from Project 7). (**B**) ClustalW alignment of expected (shown as “correct”) and incorrect (shown as “#720”) sequences. The region from splice acceptor (SA) to *F14* are shown

Among the 53 animals that contained the desired targeted insertion of donor plasmid, 51 had accurate recombination (96.2%), and only two mice had minor inaccuracies (3.8%) in their 5’ junctions (Fig. 4A). In the offspring #720 from Project 4, the 5’ end of the *attR* sequence was missing from the 3’ region of the splice acceptor sequence, and a portion of the *attB* sequence was inserted between them (Fig. 4B). In the offspring #65 from Project 7, the “*F14-attR-F14*” sequence in the conditional^ex^ allele (floxed^ex^ allele) was duplicated (Fig. 4A). Nevertheless, we could generate the correct conditional^Δex^ allele (floxed^Δex^ allele) by repairing the inaccurate allele, such as #65 after FLP recombination. These results indicate that *i*-PITT would be a better approach for creating targeted transgenic mice because it is more accurate. Further, minor inaccuracies in recombination could be repaired using FLP recombination to generate the correct conditional^Δex^ allele.

### Efficiency of removing extra vector sequences

It is not uncommon that vector sequences also get inserted with the *i*-PITT method. Among the 53 animals generated in this work that contained the insert, 29 (55%) contained vector sequences (Table 1; Fig. 3B). The architecture of the donor plasmids allows removal of vector sequences via FLP recombination, which can be achieved in one of three ways: (1) by including *FLPo* mRNA in the injection solution, (2) by introducing *FLPo* mRNA via injection into the cytoplasm of fertilized zygotes obtained from the founder Tg mice (to remove the vector backbone in F1 offspring), or (3) by breeding the founder mice with FLP Tg mice [27]. The first approach saves time and resources. We showed that vector sequences can be excluded in 84% (65/77) of offspring using the second approach (Table 2; Fig. 3C, Supplementary Fig. 3).

### Conditional expression of transgene

**Fig. 5:**
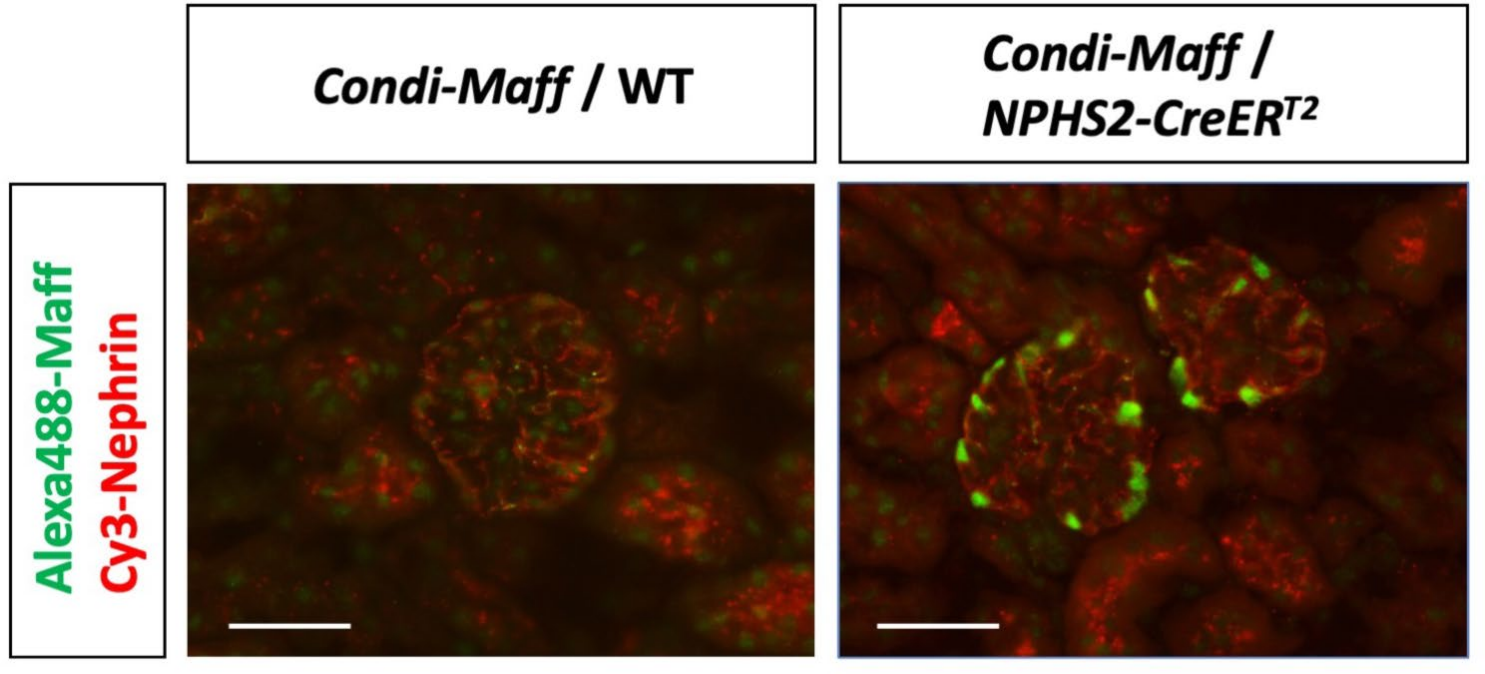
Podocyte-specific expression of Maff protein in *Condi-Maff* mouse. Representative immunofluorescence micrographs of kidney sections from *Condi-Maff* mice (without [left] or with [right] *NPHS2-CreER*^*T2*^ transgene) after administration of tamoxifen, stained with antibodies against Maff (green) and Nephrin (red). Scale bars = 50 μm

The main goal of this work was to enable insertion of constructs containing floxed cassettes using the PITT method. Given our experience with improved PITT (*i*-PITT) that the combination of integrases (PhiC31) and recombinases (Cre) significantly enhances efficiency [15], we reasoned that excluding Cre and adding FLP instead in *i*-PITT approach should achieve insertion of floxed cassettes. We successfully achieved this by developing Tg mice containing a floxed allele, targeted to the *Rosa26* locus. Conditional expression of GOIs was confirmed by mating the *Condi-Maff* Tg mouse line with *NPHS2-CreER*^*T2*^ which enables podocyte-specific *CreER*^*T2*^ expression. In the kidney of double Tg mice (*Condi-Maff/NPHS2-CreER*^*T2*^), Maff protein was expressed in podocytes after administration of tamoxifen. This confirms that the Tg mouse generated in this study elicit Cre-mediated conditional expression function as intended (Fig. 5).

## Discussion

This study represents further development of targeted transgenesis technology in mice to promote reproducible gene expression using site-specific recombination and integrase systems [14, 15]. We previously demonstrated that simultaneous use of multiple recombination systems (for example, Cre and PhiC31) can improve insertion efficiency [15]. Transgenic animals containing Cre-*LoxP*-based conditional gene expression cassettes are widely used in biomedical research. However, because Cre cannot be used for insertion of the *LoxP*-containing cassettes, we used the combination of PhiC31 and FLP to insert the construct containing *LoxP* sequences. The combination of these recombinant systems had an average insertion efficiency of 13.7% (a range of 6.9–18.0% among 11 different projects), compared to 10 to 30% (up to 62%) when Cre and PhiC31 were used together [15]. Among the 53 Tg mice generated, 51 (96.2%) were correctly recombined and only 2 (3.8%) were incorrectly recombined. Inaccurate recombination events occur in almost all genetic engineering methods including the CRISPR-based approaches [28–30]. Considering that insertions through inaccurate recombination occur more commonly using CRISPR approaches, *i*-PITT approach offers as better approach. As a general practice, it is necessary to confirm the accuracy of the insert by sequencing the junctions and confirm that the GOI expresses as expected.

Introduction of FLP in the *i*-PITT microinjection step enabled the removal of the extra sequence of the plasmid donor that gets inserted into the mouse genome. In fact, the extra sequence was successfully removed in half of the targeted founder mice. In addition, we could easily get rid of the extra sequence (if it was remained in some founder mice) by reinjection of *FLPo* mRNA. Co-injection of *FLPo* in *i*-PITT step will have the advantage of not only saving the time and effort of injecting *FLPo* to remove the extra sequence, but it also allows the creation of a strain (F1) by mating with FLPe Tg mice [27].

CRISPR-based approaches, which are widely adapted, use different types of donor DNAs such as ssDNA [31], plasmid DNA [32], or AAV vectors [33]. Although efficiency of CRISPR-based approaches, particularly using ssDNA donors, are generally high, inaccurate insertions such as missing fragments or duplication of some segments are more frequent when the size of the insert is longer than a few kilobases [34]. When plasmid DNA is used in CRISPR approaches, it is known to insert relatively accurately, but the efficiency is often lower than or comparable with that of the *i*-PITT method [35]. In addition, some vector backbones also get inserted using the CRISPR approach [36]. The homology arms used are often long in CRISPR-based approaches, which makes construction of plasmid donors time-consuming and in some cases makes it challenging to genotype [35]. Also, some loci are generally hard to amplify (for example *Rosa26* which is GC rich). Recently, a knock-in method using AAV vectors called CRISPR-READI has been reported to be very efficient [33]. However, the packaging limit of AAV vectors is only up to about 5 kb including homology arms. On the other hand, up to 15 kb sequences can be inserted using *i*-PITT. In addition, the insertion-junctions are invariably accurate, and the insertion fragments are almost always fully intact. The fact that all but one of the inserts in this experiment were larger than 4 kb suggests that these Tg cannot be produced by knock-in using AAV.

### Electronic supplementary material

Below is the link to the electronic supplementary material.

Supplementary Material 1

Supplementary Material 2

Supplementary Material 3

Supplementary Material 4

Supplementary Material 5

Supplementary Material 6

Supplementary Material 7

## Data Availability

All data generated and analyzed in this work are available in this published article and Supplementary data. Plasmids constructed in this study are available from Addgene (www.addgene.org) or the corresponding author on reasonable request. The *Condi-Maff* mice (C57BL/6-Gt(ROSA)26Sor < tm2(CAG-Egr1)Motoj>) produced and analyzed in this study are available from RIKEN BRC (RBRC11275).
