# Supplementary figures and images for: Targeted insertion of conditional expression cassettes into the mouse genome using the modified i-PITT
Source: BMC Genomics. 2024 Jun 5;25:568. doi: 10.1186/s12864-024-10250-0 (PMC11155135; doi:10.1186/s12864-024-10250-0)

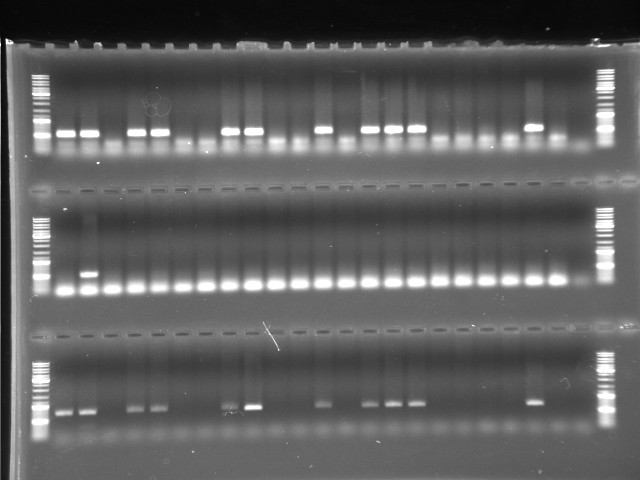

Supplement: Supplementary file 1 — Supplementary Material 1 [file 12864_2024_10250_MOESM1_ESM.tif]

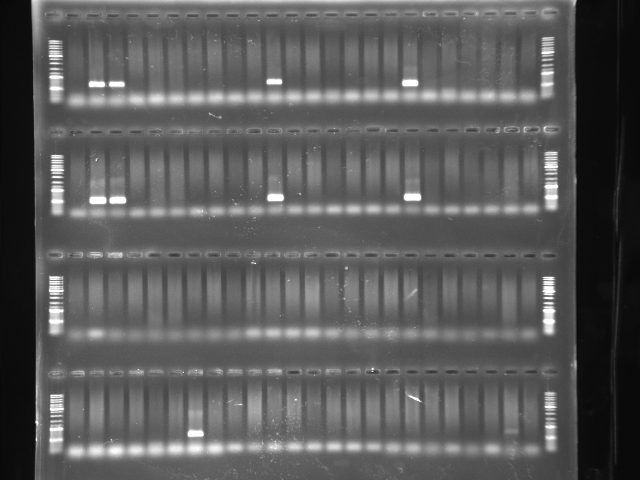

Supplement: Supplementary file 2 — Supplementary Material 2 [file 12864_2024_10250_MOESM2_ESM.tif]

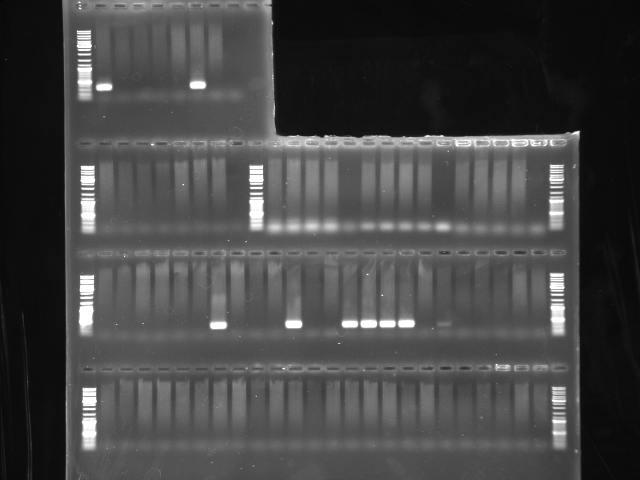

Supplement: Supplementary file 3 — Supplementary Material 3 [file 12864_2024_10250_MOESM3_ESM.tif]

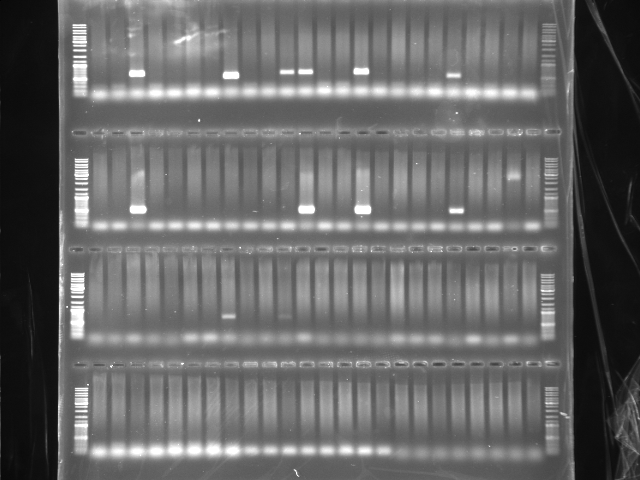

Supplement: Supplementary file 4 — Supplementary Material 4 [file 12864_2024_10250_MOESM4_ESM.tif]
