# Supplementary material for: Targeted insertion of conditional expression cassettes into the mouse genome using the modified i-PITT
Source: BMC Genomics. 2024 Jun 5;25:568. doi: 10.1186/s12864-024-10250-0 (PMC11155135; doi:10.1186/s12864-024-10250-0)
Supplement: Supplementary file 7 — Supplementary Material 7 [file 12864_2024_10250_MOESM7_ESM.pdf]

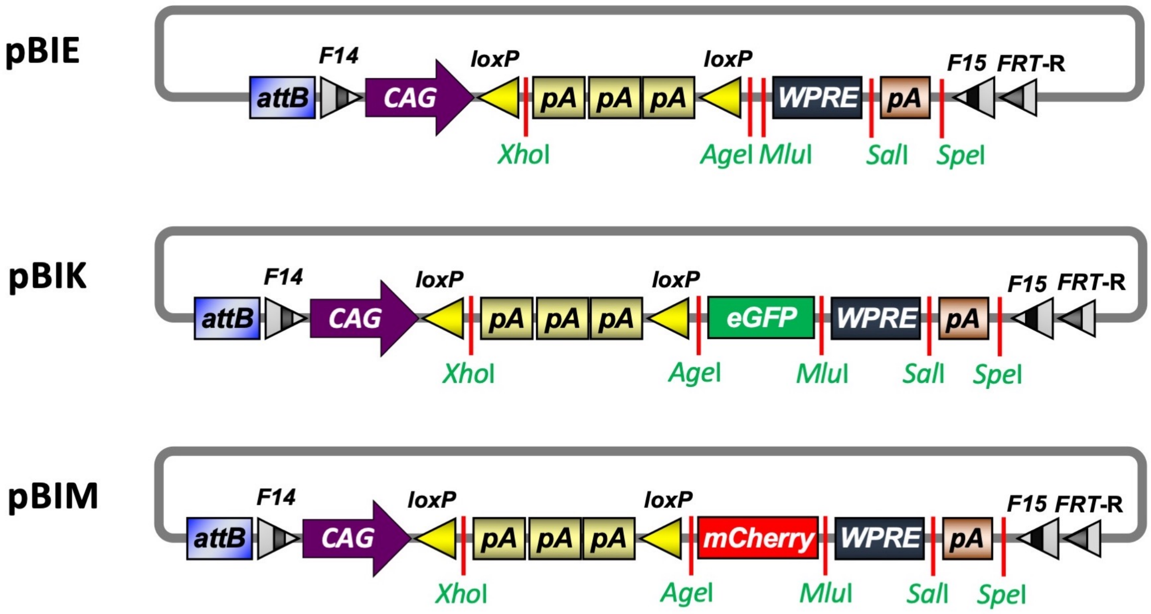

Supplementary Fig. 1. The general structure of the donor vectors used in this study. Gene of interest (GOI) can be inserted into suitable restriction site. F14, F15, FRT-R: mutant FRTs. WPRE: woodchuck hepatitis virus posttranscriptional regulatory element. pA: polyadenylation signal sequence. CAG: hybrid construct consisting of the cytomegalovirus enhancer fused to the chicken beta-actin promoter. attB: attachment sites for PhiC31 integrase.

A

AT1054.TIF

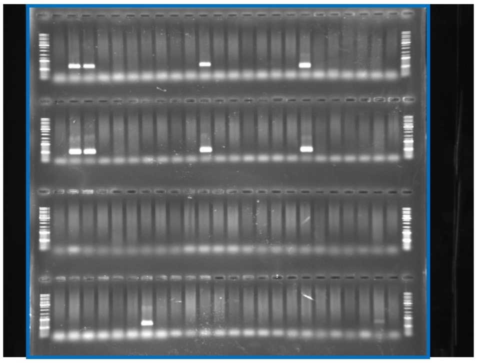

C

AT1061.TIF

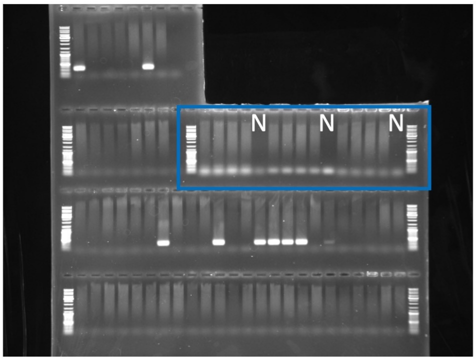

B

AT1058.TIF

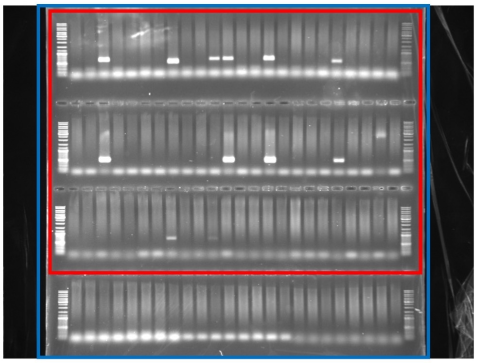

Supplementary Fig. 2. Raw data of electrophoresis images of PCR genotyping of offspring for Project 9 (mice numbers 1 to 68, see **Table 1**) using three primer sets shown in **Figure 3** (enclosed by the blue squares in A to C). (A) The first three rows from the top of the gel show genotyping results for mice numbers 1 to 24 and the fourth row for mice numbers 25 to 32. (B) The first three rows from the top of the gel show the genotyping results for mice numbers 33 to 56, and the fourth row shows the genotyping results for mice numbers 57 to 64. The area enclosed by the red square corresponds to the electrophoresis image shown in **Figure 3B**. (C) The blue boxes show the genotyping results for mice numbers 65 to 68. N: Negative control.

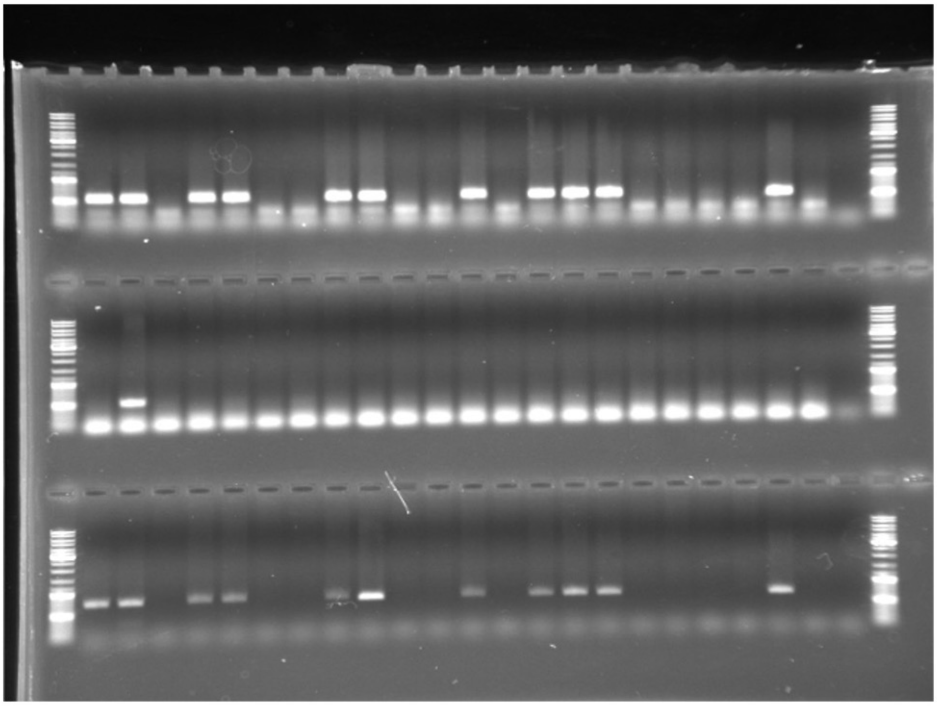

AT0446.TIF

Supplementary Fig. 3. Raw data of electrophoresis image of PCR genotyping of 22 pups (mice numbers 345 to 366) obtained by injecting FLPo mRNA into fertilized eggs derived from offspring #56 with extra vector sequence obtained in Project 6 (**Table 2**). This gel image corresponds to the electrophoresis image shown in **Figure 3C**.
